# Physical Properties and Color Stainability by Coffee and Red Wine of Opaque and High Translucency Zirconia Dental Ceramics after Hydrothermal Degradation

**DOI:** 10.1155/2022/1571729

**Published:** 2022-05-21

**Authors:** Ana Lúcia Nascimento Oliveira, Carlos Nelson Elias, Heraldo Elias Salomão dos Santos, Claudinei dos Santos, Ronaldo Sergio de Biasi

**Affiliations:** ^1^Instituto Militar de Engenharia, Pr. General Tiburcio 80, Rio de Janeiro 22290-270, Brazil; ^2^Universidade do Estado do Rio de Janeiro, Rodovia Presidente Dutra, km 298, Polo Industrial, Resende, Rio de Janeiro 27537-000, Brazil

## Abstract

The objective was to evaluate the bending strength, phase transformation, roughness, and color stainability by coffee and red wine of opaque and high translucency yttria-stabilized zirconia before and after hydrothermal degradation in saline solution or oral mouthwash. Presintered zirconia blocks with medium opacity (ZrO_2_-3 mol. % of Y_2_O_3_) designed as ZrOp and high translucency zirconia (5.2 mol. % of Y_2_O_3_) designed as ZrTrans were used. Specimens (*n* = 80/group) were cut and sintered at 1500°C for 2 h. The specimens were hydrothermally degraded in an autoclave (134°C–1.8 kg/cm^2^) for 20 h in saline solution (0.5 g/L) and oral mouthwash solution (0.02% sodium fluoride, without alcohol and with 21.6% alcohol). After hydrothermal degradation, the samples were immersed in containers with coffee or red wine for 14 days to determine their color stainability. The results showed that the ZrOp had a higher bending strength than the ZrTrans before and after hydrothermal aging. In ZrOp and ZrTrans, the roughness increased after hydrothermal degradation. ZrOp samples had a higher Ra roughness than the ZrTrans samples. Roughness did not change after immersion in coffee or red wine. The X-ray diffraction (XRD) results showed that ZrOp samples underwent a tetragonal to monoclinic phase transformation, while ZrTrans samples were unchanged. Both ZrOp and ZrTrans samples changed color after immersion in coffee and red wine.

## 1. Introduction

Restorative and prosthetic techniques in dentistry have undergone significant changes in recent years with increasing patient demands. Until the last decade, during dental treatment, patients were only concerned with functional rehabilitation. Currently, patients wish that dental treatments should also provide good esthetic results. To meet the new requirements, advanced dentistry techniques and restorative materials have been developed [1, 2]. Among the several changes, it is worth mentioning the use of metal-free ceramic materials. Dental ceramics have a more natural, translucent look than metal-ceramic restorations [3, 4], but the mechanical properties and the color are susceptible to degradation in the oral environment. In the oral environment, materials are in contact with acidic and basic solutions and are subject to temperature variations and compressive, torsion, and shear loads. This environment facilitates the degradation of materials, especially in the case of tetragonal zirconia ceramics, 3Y-TZP [5].

Tetragonal zirconia partially stabilized with 3% mol yttria (3Y-TZP) has a flexural strength of 1000 to 1300 MPa [4–6], which is larger than that of traditional ceramics. One of its drawbacks is the possibility of long-term mechanical degradation in the oral environment, known as low-temperature hydrothermal degradation (LTHD), which occurs through spontaneous and progressive transformation of the metastable tetragonal phase to a monoclinic phase [7–10]. In addition to hydrothermal degradation, the prostheses may undergo color changes due to contact with oral fluids and ingested drinks. The phase transformation spreads through the grain contours and leads to the development of cracks that may compromise the integrity of the prosthesis [11].

Details of processes involved in phase transformation and the kinetics of zirconia degradation are described by Chevalier et al. [11]. The phase transformation from tetragonal to monoclinic zirconia is of the martensitic kind transformation and involves the simultaneous cooperative movement of atoms over distances less than an atomic diameter.

The phase transformation of zirconia is slow at room temperature but can be accelerated by heat treatment between 200 and 300°C. Kim et al. [12] studied the influence of phase transformation of zirconia on microstructure and flexural strength. Specimens of Y-TZP were kept in an autoclave for 10 h at a temperature that increased from 75°C to 200°C in increments of 25°C. The flexural strength increased with temperature for temperatures up to 125°C and the volume fraction of the monoclinic phase increased with temperatures up to 200°C.

Silva et al. [13] studied the degradation of different types of zirconia and cladding ceramics and observed that there was a partial phase transformation of monoclinic to tetragonal in conventional zirconia, but not in high translucency zirconia. The authors also found that hydrothermal aging did change the optical properties. However, mechanical degradation alone or associated with hydrothermal aging reduced the reflection factor and increased opacity, especially in high translucency zirconia. Fonseca et al. [14] studied the degradation kinetics of highly translucent zirconia at 134°C. They found that degradation begins on the surface, with rapid tetragonal-to-monoclinic phase transformation, and stabilizes after a period of 26 h. They also found that the volume fraction of the monoclinic phase was higher in the surface layers. The authors report that the surface degradation of zirconia can show characteristic features such as displacement of zirconia grains, highlighting the polishing lines of the specimens, and the presence of artifacts originating from grains that have failed during *t* ⟶ *m* transformation. Conventional zirconia showed a higher level of surface degradation than highly translucency zirconia.

Mukaeda et al. [15] studied the degradation of 3Y-TZP zirconia in artificial saliva for several pH values and in the presence of fluorides. They observed that media with acidic pH and fluorides lead to a higher degree of degradation due to the formation of HF, which in turn form YF_3_ by removing Y ions from zirconia. This behavior is reversed when zirconia is exposed only to hot water, indicating the influence of pH and fluoride. The authors found that the degree of susceptibility to degradation may be related to grain size since zirconia with nanometric grains was more susceptible to degradation than zirconia with micrometric grains.

Amarante et al. [16] studied the effect of surface roughness on the flexural strength of Y-TZP specimens stabilized with 3 mol. % and 5 mol. % of Y_2_O_3_. For the analysis, the specimens were polished or sandblasted with alumina (Al_2_O_3_). X-ray diffraction analysis showed that the tetragonal phase was present in all specimens. It was found that the surface of Y-TZP with 5 mol % of yttria blasted with alumina had a roughness Ra parameter 16 to 26 times larger than a polished surface. The Ra parameter of Y-TZP with 3% mol of yttria was 38 times larger than that of Y-TZP with 5% mol of yttria. Blasting of 3 mol% Y_2_O_3_ ZrO_2_ and 5 mol% Y_2_O_3_ ZrO_2_ zirconia reduced the flexural strength by 23% and 37.5%, respectively.

Consumption of colored beverages such as coffee, wine, coke, and tea affects the color stability of composite resin dental restorations. Coffee has a high ability to change the color of natural teeth. Red wine can affect the color stability of composite resin due to its red color [17]. Ban [18] analyzed the discoloration of hybrid resins for CAD/CAM immersed in an acidic red solution (rhodamine) and red wine. The hybrid resins show noticeable discoloration in red wine.

The literature data [13–16] show that the chemical composition, grain size, and processing of zirconia influence its hydrothermal degradation. With the hydrothermal degradation, the surface roughness is modified, which induces the adhesion of the biofilm on the surface of the dental prostheses. Degradation and discoloration of dentures are more critical in patients with poor oral hygiene.

The objective of the present work is to evaluate and compare the hydrothermal degradation of 3Y-TZP and 5Y-TZP in terms of phase transformation, mechanical properties, roughness, and color stainability after immersion in a beverage (coffee and red wine).

## 2. Materials and Methods

### 2.1. Materials

Two commercially available presintered blocks (40 × 19 × 6 mm) of yttria-stabilized tetragonal zirconia (Y-TZP) ceramics with two concentrations of yttrium oxide (Y_2_O_3_) were used in this work. The first group (ZrOp) of presintered blocks was produced from the 3Y-TZP commercial powder 3YSB-E® (Tosoh, Japan), has conventional translucency, and is indicated for extensive dentistry prostheses. The second group (ZrTrans) of presintered blocks was produced from the commercial powder Zpex Smile® (Tosoh, Japan), which has high translucency and is indicated for restoration of anterior teeth. The specifications of these materials are summarized in Table 1.

Fonseca et al. [14] evaluated the transmittance of visible light for four types of presintered zirconia blocks used in dentistry (3Y-SBE, Zpex, Zpex-4, and Zpex-Smile). They analyzed the simultaneous influence of sintering temperature (from 1450 to 1560°C), chemical composition (%Y_2_O_3_), density, and thickness (1.0, 1.3, 1.6, and 2.0 mm) in zirconia transmittance. They showed that samples made with powder Zpex Smile® have higher transmittance. The transmittance of 3Y-SBE, Zpex, and Zpex-4 decreases approximately linearly with the specimen thickness, whereas zirconia Zpex-Smile has a sublinear decrease, which is expected due to the optical isotropy of the cubic phase. Based on Fonseca et al. [14] results, in the present work, we named the 5Y-TZP (5.2 mol. % of Y_2_O_3_) as high translucency zirconia (ZrTrans group). The presintered blocks were supplied by ProtMat Materiais Avançados (Juiz de Fora, Brazil). The compaction pressure and presintering temperature cannot be disclosed due to confidentiality reasons.

### 2.2. Specimen Processing

Eighty zirconia specimens of each type of presintered zirconia blocks (ZrOp and ZrTrans) were cut and submitted to subsequent grinding, surface polishing, and sintering. The sintering was performed in an NBD 1700°C furnace (Nobody Materials Science and Technology Co., LTD, China) at 1500°C for 2 h, using a heating and cooling rate of 5°C/min.

After sintering, the sequence of characterization and processing of the specimens was the following:

- Characterization after sintering: density, surface roughness, phase identification (X-ray), color, 4-point bending, and grain size;
- Hydrothermal degradation in an autoclave;
- Characterization after hydrothermal degradation: surface roughness, phase identification (X-ray), color, and 4-point bending;
- Beverage immersion followed by surface roughness and color measurement.

After sintering, the samples suffered shrinkage (25 × 1.5 × 2 mm). The samples were ultrasonically cleaned and separated into two subgroups. The samples in the first subgroup (*N* = 20/zirconia type) were characterized (density, surface roughness, phase identification (X-ray), color, 4-point bending test, and grain size) as-sintered state. The samples in the second subgroup (*N* = 60/zirconia type) were immersed in three solutions (saline solution 0.5 g/L (*N* = 20), mouthwash solution with 0.02% sodium fluoride without alcohol mouthwash solution (*N* = 20), and mouthwash solution with 0.02% sodium fluoride and 21.6% alcohol (*N* = 20) for hydrothermal degradation followed by characterization).

The same five sintered samples from each type of zirconia were submitted in the sequence of tests: Archimedes´ principle for density calculus, surface roughness measurement, phase identification by X-ray diffraction, color measurement, and grain size measurement.

Fifteen sintered samples from each zirconia blocks types were used for 4-point bending testing. Considering that according to technical standard (ASTM C1161-13: Standard Test Method for Flexural Strength of Advanced Ceramics at Ambient Temperature), the sample size for the 4-point bending test was 25 × 2.0 × 1.5 mm, and during sintering, a shrinkage of the order of 25% occurs, and the samples were cut with approximately 27 × 3.0 × 2.0 mm.

For hydrothermal degradation, the samples were heated at 3°C/min up to 134°C and at a pressure of 1.8 kg/cm^2^. One group of samples (*N* = 20) was kept as sintered. The hydrothermal degradation was performed for 20 h, four times longer than the required times adopted in ISO 13356-15 standard [19].

Three different environments were used for hydrothermal degradation:

  ND: not subjected to hydrothermal degradation (*N* = 20).
  SS: immersion in saline solution 0.5 g/L (*N* = 20).
  F: immersion in oral mouthwash solution (0.02% sodium fluoride without alcohol) (*N* = 20).
  FA: immersion in oral mouthwash solution (0.02% sodium fluoride and 21.6% alcohol) (*N* = 20).

With the purpose to evaluate the effect of beverage on roughness and color change, after hydrothermal degradation, the samples were immersed in coffee or red wine at 39°C for 14 days. Considering the type of zirconia and the kind of hydrothermal degradation, the samples after sintering were divided into 8 groups (*N* = 5/group).  Table 2 shows the groups, names, type of zirconia, and degradation environment.

### 2.3. Characterizations

The bulk density was measured in distilled water using Archimedes´ principle. The relative density (*N* = 5/group) was calculated by correlating the bulk density with the theoretical density (*ρ*_*T*_ = 6.05 g/cm^3^). The same samples were used for X-ray diffraction, roughness measurement, color, and finally grain size measurement.

The identification of the crystalline phases (*N* = 5/group) was performed using X-ray diffractometry (XRD) before and after hydrothermal degradation. The instrument was a PANalytical X'Pert PRO diffractometer equipped with a Cu-K*α* radiation source, with an angular step of 0.02°. HighScore Plus software version 3.0e (3.0.5) 2012 from PANalytical was used to determine the crystalline phases. The diffractogram data were compared to those of the ICDD (International Centre for Diffraction Data) and COD-2012 (Crystallography Open Database) PDF2-2004 databases. The Rietveld method was employed to quantify the phases and analyzed the crystal structures.

The surface roughness (*N* = 5/group) of the specimens was measured before and after degradation using a noncontact coherence scanning interferometry (CSI) technology model NewView 7100 (Zygo Corporation, Connecticut, USA). Five measurements per specimen were performed. Three roughness parameters were measured (Ra, Rz, and PV). Although the parameter Ra is the most analyzed in the literature, our previous results showed that among the various roughness parameters, the parameters Ra, Rz, and PV are those that exert the greatest influence on dental implant properties. Three roughness areas per sample were measured.

For the grain size measurement, a thermal treatment was carried out (1400°C for 15 min at a rate of 20°C/min) to reveal the zirconia grain boundary. The samples were covered with platinum coating for imaging in a scanning electron microscope (FEI Quanta FEG250, Hillsboro, Oregon, USA). An intercept procedure according to ASTM E112 and Feret's diameter method through automatic image analysis using Image-Pro Plus 6.0 software (Media Cybernetics Inc.) was used to measure the grain size. This procedure was used previously [7].

**Figure 1:**
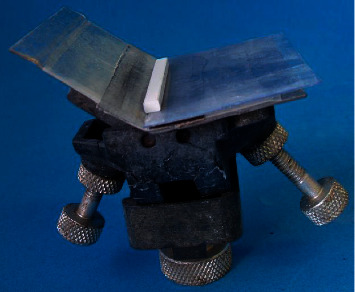
Setup to prepare the specimen 4-point bending chamfer on the edges.

The ASTM C 1161 standard also defines the presence of a chamfer or rounding of the edges of the 4-point bending specimens. In the present work, the first option was chosen. The four long edges of each specimen were uniformly chamfered at 45°. To perform the chamfer on the edges, an adjustable table with 45° was adapted (Figure 1).

Four-point bending tests (*N* = 15/group) were performed using a universal mechanical testing machine EMIC model DL-10000 (EMIC Equip. Sist., Instron Group; São José dos Pinhais, Brazil). A loading rate of 0.2 mm/min and a cell with 1 kN was used. The bending test was performed following the recommendations of the ASTM C 1161-08 standard [20].

A custom four-point flexure fixture having a support span (*L*) of 20 mm and a loading span of 10 mm was used. The width (*w* = 8 mm) and thickness (*t* = 1.5 mm) of each specimen were measured to determine the flexural strength (*σ*) according to the following equation:

(1) $$\begin{array}{c} \sigma =\frac{3PL}{4wt^{2}}, \end{array}$$

where *P* is the maximum load recorded during testing.

Weibull statistical analysis was performed on the results of the bending test.

Before (ND) and after hydrothermal degradation (SS, F, and FA), three bar-shaped samples from both zirconia types (ZrOP and ZrTrans) were immersed in containers with coffee or red wine for 14 days to determine the color stainability. After immersion, the samples were washed with ultrasound in distilled water for 3 min and the color spectrum was analyzed. The color of the sample still moist to simulate the oral environment was identified using an Easyshade Vita V (VITA Zahnfabrik, Bad Säckingen, Germany) digital spectrophotometer and the VITA Toothguide 3D-MASTER scale. The color of the specimens was measured against a black background using a standard DayLight ICI (International Commission on Illumination).

The results obtained with Easyshade V make it possible to interpret the color information in the VITA 3D-Master scale system. The Easyshade Vita spectrophotometer is based on the CIELab system on a black background. Each sample was chromatically measured three times, and the average values were calculated. Color quantification was expressed according to *L*^∗^, *a*^∗^, and *b*^∗^ color parameters. The parameter *L*^∗^ is the lightness coefficient, ranging from black (=0) to white (=100); *a*^∗^ is the shade of redness on positive values and greenness on negative values; and *b*^∗^ indicates yellowness on positive values and blueness on negative values. Color difference Δ*E* between the control group (only sintered sample and hydrothermal aging) and the color of zirconia after hydrothermal aging before and after immersion in coffee or red wine was calculated as follows:

- Δ*L*^∗^ = *L*^∗^ control − *L*^∗^ experimental
- Δ*a*^∗^ = *a*^∗^ control − *a*^∗^ experimental
- Δ*b*^∗^ = *b*^∗^ control − *b*^∗^ experimental

The color change (Δ*E*) was calculated by the following equation:

(2) $$\begin{array}{c} \Delta E={\left[\left(\Delta L^{*}\right)2+{\left(\Delta a^{*}\right)}^{2}+{\left(\Delta b^{*}\right)}^{2}\right]}^{1/2}. \end{array}$$

Color change (Δ*E*) was classified into 3 clinically relevant intervals as follows: Δ*E* < 1 (undetectable color change), 1 < Δ*E* < 3.3 (acceptable color change), and Δ*E* > 3.3 (unacceptable color change).

### 2.4. Statistical Analysis

For the statistical analysis, the null hypothesis was that the zirconia type (opaque and high translucency) and immersion in coffee and red wine do not affect the color stability of the ceramics. The ZrOp samples made with 3Y-TZP sintered and not subjected to hydrothermal degradation were the control group.

Considering that several tests were performed to characterize the samples, the statistical analysis of the results was different according to the tests. The 4-point bending strength results were analyzed by the Weibull methodology. The statistical analysis for density, grain size, and roughness was performed with one-way ANOVA followed by Bonferroni and Tukey tests at a significance level of 0.05. The statistical analysis of colors was performed using the Origin-7 software (OriginLab Corporation, Northampton, MA–USA).

Descriptive statistics (mean and standard deviation) values were calculated for each CIE *L*^∗^, *a*^∗^, and *b*^∗^ parameter. The Shapiro–Wilk test was applied to assess the normality of the distribution. A one-way nonparametric analysis of variance test (Kruskal–Wallis ANOVA) and the Tukey test were applied to determine whether significant differences existed among the groups. Mann–Whitney test was used as a post hoc. The statistical differences in color (Δ*E*) among samples before and after immersion in coffee or red wine were compared using one-way ANOVA and the Tukey test. Statistical analysis of colors was performed using the Origin-7 software (OriginLab Corporation, Northampton, MA, USA) and SPSS Statistics for Windows (IBM Corp., Chicago, IL, USA).

## 3. Results and Discussion

The results of relative density of sintered samples were superior to 98.5% of theoretical density for both groups (ZrOp and ZrTrans), and no significant statistical difference was observed between both groups.

**Figure 2:**
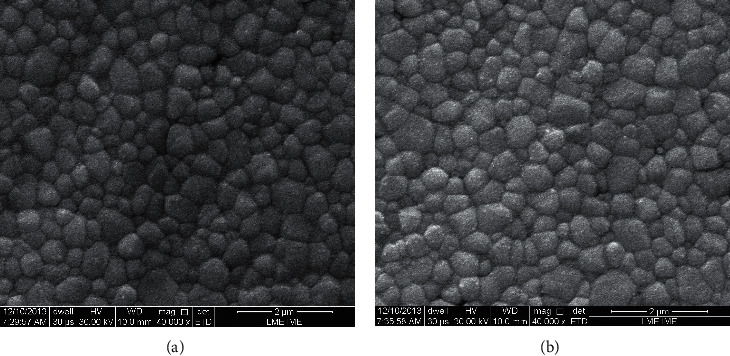
SEM micrographs of sintered samples: (a) ZrOp (ZrO_2_-3 mol. % Y_2_O_3_) and (b) ZrTrans (ZrO_2_-5 mol. % Y_2_O_3_).

Figure 2 shows the microstructures of the ZrOp (control group) and ZrTrans samples after sintering and before hydrothermal degradation. The samples of the ZrTrans sample group (5.2 mol. % Y_2_O_3_) had a higher grain size (1.6 ± 0.8 µm) than the ZrOp group (0.8 ± 0.3 *µ*m).

**Figure 3:**
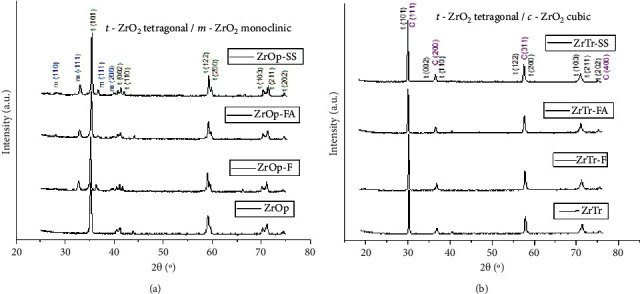
XRD patterns of sintered and hydrothermal degraded^∗^ for 20-h zirconia ceramics: (a) ZrOp and (b) ZrTrans. SS: immersion in saline solution. FA: immersion in oral mouthwash sodium fluoride solution with alcohol. F: immersion in oral mouthwash sodium fluoride solution without alcohol.

Figure 3 shows the XRD diffractograms of different groups of sintered zirconia before and after hydrothermal degradation (HD) with immersion in different liquids. The phases detected were tetragonal zirconia, *t-*ZrO_2_ (space group P42/nmc); monoclinic zirconia, *m-*ZrO_2_ (space group P21/c); and cubic zirconia, *c-*ZrO_2_ (space group Fm-3m). Furthermore, Table 3 shows the percentage of crystalline phases of zirconia ceramics before and after hydrothermal degradation (LTHD) in different solutions.

Analysis of the XRD results showed that the ZrOp and ZrTrans zirconia after sintering did not present detectable fractions of the monoclinic phase (ZrO_2_-m) on the surface. The ZrOp group has only the tetragonal phase (ZrO_2_-t), and the ZrTrans group has a 35% tetragonal phase and 65% cubic phase (ZrO_2_-c). A joint analysis of the X-ray diffraction results with micrograph analysis (Figure 3(a)) shows that the zirconia contains 3 mol. % Y_2_O_3_, which has a tetragonal phase, a microstructure with smaller grain sizes and small size variations. The cubic phase of the ZrTrans samples facilitated the abnormal grain growing (Figure 3(b)).

The high translucency zirconia (ZrTrans) did not show the total transformation of t-m phases after hydrothermal degradation for 20 h in different aqueous solutions. The ZrTrans group had 36% of the untransformed tetragonal phase. A similar was observed by Amarante et al. [21], although in the present work a different hydrothermal degradation test procedure was adopted. Amarante et al. [21] used the autoclave immersion recommended by the ISO 13356 standard (5 h, 134°C, 2 bar, and deionized water). The result of the present work shows that increasing the degradation time, as well as changing the immersion liquid, did not influence the ZrTrans phase transformations of zirconia. The microstructure has stability, and the residual tetragonal phase does not undergo martensitic transformation t-m.

The 3Y-TZP ZrOp samples, whose main crystalline phase was the tetragonal phase, showed considerable hydrothermal degradation in the liquid environments. The monoclinic phase content was 21% in the ZrTrans-FA group and 27.3% in the ZrTrans-SS group. The highest (30.8%) martensitic transformation t-m was observed for the group of samples ZrTrans-F.

Mukaeda et al. [15] evaluated the degradation of 3Y-TZP ceramics immersed in an acidic and fluoride environment. They concluded that the ZrO_2_ ceramics stabilized with Y_2_O_3_ can degrade in the presence of acid pH and fluorides, and the microstructure influences the degradation. An explanation for this behavior would be the formation of YF3, which induces the tetragonal-monoclinic phase transformation. Marcos et al. [22] showed that in both aqueous and ethyl environments, the surface of the zirconia becomes more acidic than the environment.

The ceramic surface charges are generated by the adsorption of ions from the solvent on the surface of the zirconia. The contact of some ceramic materials with water induces water decomposition, and H^+^ and OH^−^ radicals are strongly linked to the surface. When the surface is more acidic than the medium, the surface has negative charges, and the zeta potential is negative. When the surface is more basic than the medium, the zeta potential is positive.

In cases where the surface charges are generated by the adsorption of ethoxide ions from the solvent (bound by the surface zirconium and the oxygen of the ethoxide group) on the surface of the zirconia, it appears that when the bonding of the ethoxide ion and OH radicals strongly linked to the surface of the zirconia (the water decomposes), there is a stretching of the bonding of the OH group attached to the surface of the zirconia and the disappearance of vibrations.

### 3.1. Surface Roughness Morphology

**Figure 4:**
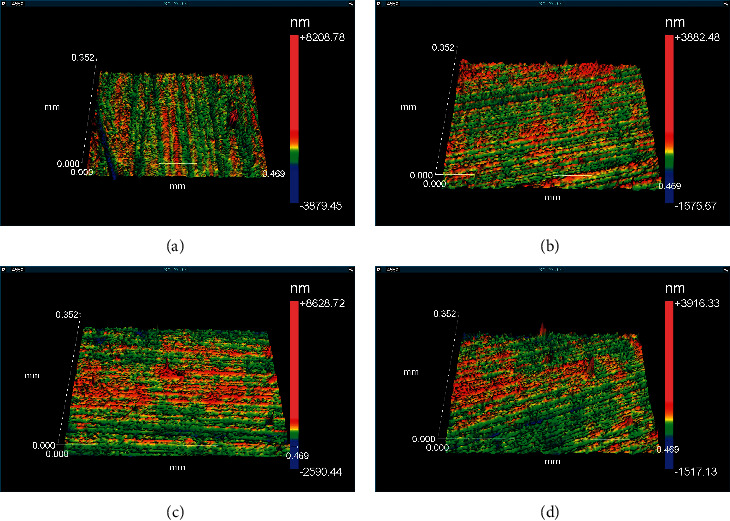
Surface morphology of ZrOp after hydrothermal degradation. (a) ZrOp-control, (b) ZrOp-SS, (c) ZrOp-FA, and (d) ZrOp-F. Image obtained using Zygo optical profiler.

**Figure 5:**
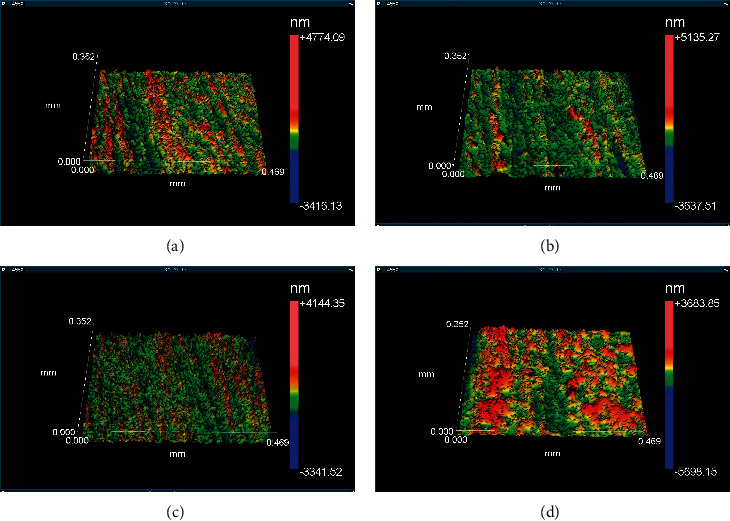
Surface morphology of ZrTrans samples after hydrothermal degradation. (a) ZrTrans control group, (b) ZrTrans-SS, (c) ZrTrans-FA, and (d) Zr-Trans-F. Image obtained using Zygo optical profiler.

The sample surface morphologies before and after hydrothermal degradation are shown in Figures 4 and 5. All images have been obtained by interferometry on the Zygo profilometer.

The results of surface roughness measurements are shown in Table 4. Specimen roughness parameters were quantified before and after hydrothermal degradation in an autoclave (134°C–1.8 kg/cm^2^) for 20 h in different environments (saline solution (0.5 g/L) and oral mouthwash (0.02% sodium fluoride, without alcohol and with 21.6% alcohol). After hydrothermal degradation, the ZrOp samples had a higher Ra roughness parameter than the ZrTrans samples. This result shows that zirconia ZrTrans has greater stability than zirconia ZrOp.

Analyzing together the hydrothermal degradation and the roughness for the YTZP group, we verified that the surface roughness was higher for the degraded group, which presented tetragonal-monoclinic transformation (*t* ⟶ *m*). This result indicates that there was a diffusion of liquid to the inner layers, which, in turn, induced the phase transformation and increased the surface roughness. The conventional zirconia (ZrOp) presents greater degradation than the translucency zirconia (ZrTrans).

After hydrothermal degradation and immersion in coffee or red wine to analyze the possible color change, the roughness of the same samples was measured again. The zirconia roughness increased after hydrothermal degradation, but immersion in coffee or red wine did not change the roughness parameters.

ANOVA indicated a statistically significant difference in the Ra roughness parameter among all tested groups (Table 5), considering that the *F* value was greater than the *F* critical value and *P*-value >0.05. All roughness parameters of the ZrOp group were higher than the ZrTrans group. For the ANOVA analysis, the null hypothesis showed that the means of zirconia group roughness are equal, but the alternative hypothesis showed that the means of one or more selected datasets are different (Tables 5 and 6). The surface roughness of specimens from the ZrTrans-F group was different from all groups (Table 7).

### 3.2. Flexural Strength

The 4-point bending strength test results are shown in Table 8.  Table 9 shows the results of the statistical analysis.

The flexural strength of the ZrOp group was 942 MPa. After hydrothermal degradation in a different environment, the flexural strength increased. This result can be explained by surface roughness change and phase transformation. Analysis of the Rietveld refinement results complemented by the surface roughness shows that a martensitic transformation in the degraded materials occurs. Previous studies based on hydrothermal degradation using water indicate that the phase transformation can increase or decrease the flexural strength, depending on the grain extrusion intensity on the surface during the monoclinic zirconia transformation. When the phase transformation is associated with the pullout of the surface grains, the surface roughness increases, and crack nucleation on the surface occurs. In this case, degradation decreases the flexural strength.

Another possibility is martensitic transformation on the surface during hydrothermal degradation. In this case, compressive residual stress occurs due to the volume difference between the monoclinic and martensitic phases. The residual stress decreases crack growth and flexural resistance increases. This seems to be the case for the ZrO_2_ group (3 mol% Y_2_O_3_), which, despite the increase in roughness, did not suffer the pullout of the monoclinic grains. Samples from the ZrTrans group show a loss of flexural strength after degradation, and the phase transformation was not detectable by X-ray diffraction. The lower mechanical resistance of the ZrTrans group than the ZrOp group may be associated with the cubic phase present in the material and the heterogeneous grain size.

Table 9 shows the statistical analysis of the results of the bending test. Samples from different ZrOp groups did not show flexural strength statistically significant differences (the *F* value was lower than the *F* critic). Samples from different ZrTrans groups had a statistically significant difference (the *F* value was higher than the *F* critic). The flexural strength of ZrTrans samples was reduced after hydrothermal degradation.

For the Zr-Tr group, there was no significant difference between the phase volume fractions after hydrothermal degradation and bending did not influence the phase transformation. It was found that there was a reduction in the bending strength of the degraded Zr-Trans group samples. In this case, the lower strength may be related to defects on the material surface and roughness. As expected, the bending strength of the Zr-Tr group was lower than that of the Y-TZP group for all subgroups.

ANOVA showed that there is no statistically significant difference among the subgroups Y-TZP-ND, Y-TZP-SS, Y-TZP-FA, and Y-TZP-F. The F value was less than the critical F value (*P*-value >0.05). After degradation, the bending strength of Y-TZP zirconia increases. This variation was not statistically significant, being a little more pronounced for the Y-TZP-FA zirconia. Possibly, the greater bending strength is due to the higher volume fraction of the tetragonal phase after hydrothermal degradation, associated with the presence of alcohol in the fluorine composition.

Analyzing the influence of roughness on the bending strength of the two types of zirconia (ZrOp and ZrTrans), it is observed that the bending strength decreases as the roughness increases, except for the subgroups ZrOp-SS and ZrTrans-FA. For the ZrOp-SS subgroup, a hypothesis for such behavior may be due to the percentage of tetragonal phase present, around 79%, greater than the percentage of the other ZrOp subgroups.

### 3.3. Color Stainability Tests

The results of the color stainability tests after immersion in coffee or red wine for 14 days are summarized in Table 10. The indexes in Table 10 are based on the Vita Toothguide 3D-MASTER index scale. It was not possible to measure the color of nondegraded Zr-Trans samples, but after autoclave hydrothermal aging for 20 h in coffee, the color was 1M1. After hydrothermal aging for 20 h and immersion in red wine, the color was 2M2C.  Table 11 shows the mean *L*^∗^, *a*^∗^, and *b*^∗^ values (SD) for each shade specimen group.

The results of the statistical color analysis are summarized in Table 12. Shapiro–Wilk test confirmed that the values were not normally distributed. Kruskal–Wallis ANOVA found significant differences among the various restorative materials. The results of the statistical analysis showed that the null hypothesis was rejected since the zirconia surface properties change by immersion in coffee or red wine.

Due to the lack of a technical standard to perform zirconia degradation tests, the comparison of results is impaired. Researchers carry out their tests with specimens prepared in different ways and immersion environments with different compositions, pH, and temperatures. Despite this difficulty, comparisons were made between the results of this study and those available in the literature. Paravina et al. [23, 24] described and analyzed the color measurement methodology in detail.

The results obtained in the present work corroborate those obtained by Barutçugil et al. [25]. They analyzed the discoloration of three types of CAD-CAM blocks (3M Lava Ultimate (LU), GC Cerasmart (CS), and VITA Enamic) after being immersed in distilled water, red wine, and coffee for 30 days. They observed that the zirconia immersed in red wine and coffee for 1 month showed greater discoloration than those immersed in water. But Colombo et al. [26] observed that only one-week immersion in the acidic drink (Coke) did not cause a perceivable zirconia discoloration and subsequent immersion in coffee affected the color stability of all zirconia samples. The different result has been observed by Ban et al. [18], who cited that zirconia showed no changes in a lactic acid at 60°C, KOH solution at 60°C, and saline solution at 90°C. Based on comparisons of results with those obtained in the present work with those in the literature, we can conclude that zirconia may have the color change, but the results depend on the test conditions like pH environment, sequence of immersion, and type of liquid.

## 4. Conclusions

The results of the present work showed that it is important for dentists to take special care in recommending the use of zirconia and to guide their patients regarding the intake of colored drinks, especially coffee and red wine.

The results of sample characterization before and after hydrothermal aging showed that:

- After sintering, conventional translucency zirconia with 3 mol % Y_2_O_3_ (ZrOp) had only the tetragonal phase (ZrO_2_-t) and high translucency zirconia with 5 mol % Y_2_O_3_ (ZrTrans) had a tetragonal phase (36%) and a cubic phase (64%). After hydrothermal degradation, ZrTrans did not have a significant phase transformation and ZrOp underwent a tetragonal to monoclinic phase transformation.
- Before and after hydrothermal aging, ZrOp zirconia had a higher bending strength than ZrTrans.
- Sintered ZrTrans zirconia had a higher Weibull modulus than ZrOp. After hydrothermal aging, the Weibull modulus of ZrOp increased and the Weibull modulus of ZrTrans decreased.
- The surface roughness of Zr-Trans increased after hydrothermal degradation.
- Hydrothermal degradation and immersion in coffee or red wine changed the color of conventional and highly translucency zirconia.
- There was not a significant relationship between roughness and color stainability in conventional and high translucency zirconia.

## Figures and Tables

**Table 1:** Characteristics of different presintered ceramics used in this work (manufacturing data).

Designation	ZrOp (3Y-TZP - Opaque)	ZrTrans (5Y-TZP -Translucent)
Nominal percentage of Y_2_O_3_	3 mol. %	5 mol. %
Powder commercial name	3YSB-E (Tosoh corp.)	Zpex Smile (Tosoh corp.)
Chemical composition (wt.%)		
Y_2_O_3_	5.1	9.3
Al_2_O_3_	0.25 ± 0.1	0.05
SiO_2_	≤0.02	≤0.02
Fe_2_O_3_	≤0.01	≤0.01
Na_2_O	− ≤0.01	—
ZrO_2_+HfO_2_	Balance	Balance

**Table 2:** Groups' names, type of zirconia, and hydrothermal degradation environment.

Groups	Material	Degradation	Degradation environment
ZrOp-Control	3Y-TZP	No	
ZrOp-F	3Y-TZP	20 h	mouthwash with fluoride and without alcohol.
ZrOp-FA	3Y-TZP	20 h	fluoride mouthwash containing alcohol
ZrOp-SS	3Y-TZP	20 h	saline solution of 0.5 g/L
ZrTrans	5Y-TZP	No	
ZrTrans-F	5Y-TZP	20 h	mouthwash with fluoride and without alcohol.
ZrTrans-FA	5Y-TZP	20 h	fluoride mouthwash containing alcohol
ZrTrans-SS	5Y-TZP	20 h	saline solution of 0.5 g/L

**Table 3:** Percentage of crystalline phases of zirconia before and after low-temperature hydrothermal degradation in oral mouthwash sodium fluoride solution without alcohol (LTHD-F), oral mouthwash sodium fluoride solution with alcohol (LTHD-FA), and saline solution (LTHD-SS).

Experimental condition	Crystalline phases (wt. %)	ZrOp	ZrTrans
Sintered (control group)	m-ZrO_2_ (Monoclinic)	—	—
	t-ZrO_2 (_Tetragonal)	100	36.0
	c- ZrO_2_ (cubic)	—	64.0

LTHD-F	m-ZrO_2_ (Monoclinic)	30,8	—
	t-ZrO_2 (_Tetragonal)	69,2	35.0
	c- ZrO_2_ (cubic)		65.0

LTHD-FA	m-ZrO_2_ (Monoclinic)	21,0	—
	t-ZrO_2 (_Tetragonal)	79,0	35,2
	c- ZrO_2_ (cubic)	—	64,8

LTHD-SS	m-ZrO_2_ (Monoclinic)	27,3	
	t-ZrO_2_ (Tetragonal)	72,7	36,0
	c- ZrO_2_ (cubic)	—	64,0

**Table 4:** Roughness parameters of Zr-Trans and Y-TZP samples before (ND) and after degradation in different solutions. The roughness average was calculated based on the 5-sample testing and 3 measurements by sample.

Samples	Ra (*µ*m)	Rz (*µ*m)	PV (*µ*m)
ZrOp control	0.82 ± 0.16	22.6 ± 9.4	34.1 ± 19.1
ZrOp-SS	1.09 ± 0.25	25.5 ± 5.3	36.4 ± 4.8
ZrOp-FA	1.29 ± 0.08	28.2 ± 3.1	38.1 ± 5.7
ZrOp-F	1.58 ± 0.05	29.6 ± 3.3	42.8 ± 7.3
ZrTrans	0.54 ± 0.02	21.0 ± 0.1	25.7 ± 0.3
ZrTrans-SS	0.59 ± 0.13	22.3 ± 3.0	28.4 ± 6.0
ZrTrans-FA	0.54 ± 0.13	24.1 ± 8.5	33.5 ± 11.2
ZrTrans- F	0.67 ± 0.08	29.5 ± 1.1	40.7 ± 15.1

**Table 5:** ANOVA of Ra roughness after hydrothermal degradation of all groups.

	Degrees of Freedom	Sum Sq	*F* value	*P*-value	*F* critic
Model	7	1.258	22.28	1.284*E* − 10	2.866
Error	32	0.258			
Total	39	1.506			

**Table 6:** ANOVA of Ra roughness after hydrothermal degradation of all specimens of ZrOp.

	Degrees of Freedom	Sum Sq	*F* value	*P*-value	*F* critic
Model	3	0.629	20.14	1.110*E* − 5	2.734
Error	16	0.166			
Total	19	0.795			

**Table 7:** ANOVA Ra roughness parameters statistical analysis.

		ZrOp			ZrTrans			
		SS	FA	F	ND	SS	FA	F
ZrOp	ND	Y	N	Y	Y	Y	Y	N
	SS		Y	Y	Y	Y	Y	Y
	FA			Y	Y	Y	Y	N
	F				N	N	N	N

ZrTrans	ND					N	N	N
	SS						N	N
	FA							N

Y means the significant difference between groups and N means no significant difference between groups.

**Table 8:** Bending strength (MPa) and Weibull modulus of zirconia samples before and after hydrothermal degradation. The average was calculated based on the 15-sample testing.

	Flexural strength (MPa)			
	Not degraded	After hydrothermal degradation		
		FA	F	SS
ZrOp	941.9 ± 164	1056.6 ± 149	1121.5 ± 178.7	1012.8 ± 60.7
ZrTrans	439.2 ± 67	306.5 ± 43	268.9 ± 56.6	299.9 ± 51.3

	Weibull modulus (m)			
ZrOp	5.8	6.3	6.7	12.9
ZrTrans	9.0	8.9	5.2	6.2

**Table 9:** ANOVA of bending test results of Y-TZP and Zr-Trans groups.

		Degrees of Freedom	Sum Sq	Mean Sq	*F* value	Pr (>F)	*F* critic
Zr-Trans	Model	3	168822	56273.99	10.16777	5.43*E* − 05	2.866266
	Error	36	199243.6	5534.544			
	Total	39	368065.5				

Y-TZP	Model	3	176553.8	58851.28	1.774232	0.169504	2.866266
	Error	36	1194120	33170.01			
	Total	39	1370674				

**Table 10:** Sample index colors after immersion in coffee and red wine, based on the Vita Toothguide 3D-MASTER index scale.

	ZrOp		ZrTrans	
	Coffee	Red wine	Coffee	Red wine
ND	2M1C	2M1C	—	—
F	2M1C	2M2C	1M1	2M2C
FA	2M2C	2M2C	1M1	2M2C
SS	2M1C	2M1C	1M1	2M2C

^*∗*^Not measured.

**Table 11:** Comparison between color parameters of different core types. Means (SD) of CIE *L*^∗^, *a*^∗^, and *b*^∗^ for each shade (*n* = 3).

Index color	*L* ^*∗*^	*a* ^*∗*^	*b* ^*∗*^
2M1C	81.37	−0.43	13.25
2M2C	87.21	1.01	18.31
1M1	84.34	−1.12	11.23
Control sample	81.11	2,76	28.15

**Table 12:** Color difference (Δ*E*) between the control (only sintered sample) and the color of zirconia after hydrothermal aging and immersion in coffee or red wine.

	2M1C	2M2C	1M1	Control
2M1C	—	7.86	3.66	5.37
2M2C	7.86	—	7.93	6.15
1M1	3.66	7.93	—	8.16
Control	5.37	6.15	8.16	—

## Data Availability

All data are within the article.
